# Effect of acute high-intensity exercise on myocardium metabolic profiles in rat and human study via metabolomics approach

**DOI:** 10.1038/s41598-022-10976-5

**Published:** 2022-04-26

**Authors:** Lijun Wu, Jiayi Wang, Xiuhui Cao, Yue Tian, Jia Li

**Affiliations:** School of Physical Education, Shanxi University, No. 92, Wucheng Road, Taiyuan, 030006 Shanxi China

**Keywords:** Biochemistry, Physiology, Biomarkers, Cardiology, Diseases

## Abstract

Acute high-intensity exercise can affect cardiac health by altering substance metabolism. However, few metabolomics-based studies provide data on the effect of exercise along with myocardial metabolism. Our study aimed to identify metabolic signatures in rat myocardium during acute high-intensity exercise and evaluate their diagnostic potential for sports injuries. We collected rat myocardium samples and subjects’ serum samples before and after acute high-intensity exercise for metabolite profiling to explore metabolic alterations of exercise response in the myocardium. Multivariate analysis revealed myocardium metabolism differed before and after acute high-intensity exercise. Furthermore, 6 target metabolic pathways and 12 potential metabolic markers for acute high-intensity exercise were identified. Our findings provided an insight that myocardium metabolism during acute high-intensity exercise had distinct disorders in complex lipids and fatty acids. Moreover, an increase of purine degradation products, as well as signs of impaired glucose metabolism, were observed. Besides, amino acids were enhanced with a certain protective effect on the myocardium. In this study, we discovered how acute high-intensity exercise affected myocardial metabolism and exercise-related heart injury risks, which can provide references for pre-competition screening, risk prevention, and disease prognosis in competitive sports and effective formulation of exercise prescriptions for different people.

## Introduction

Exercise, especially moderate-intensity endurance training, plays an important role in preventing cardiovascular disease (CVD). Whether acute high-intensity exercise is harmful or beneficial for the heart remains controversial. Acute high-intensity exercise is a brief burst of vigorous, high-intensity (usually ≥ 85% of VO2peak) exercise^1^. It refers to increased energy consumption and changed material metabolism, which results in a decrease in ATP synthesis efficiency. At this moment, the oxygen supply of tissue, associated with an electron leakage of the mitochondrial respiratory chain, decreases, easily inducing oxidative stress damage ^2^.

Data indicate short-term (11.9 ± 2.1 min) maximum incremental exercise can significantly alter the circulating metabolome, which is beneficial for reducing CVD risk factors^3^. However, the benefits of acute high-intensity exercise need to be balanced against the potential increased risk of exercise-related adverse effects. Data also suggests the rate of cardiac arrest during acute high-intensity exercise is five times higher than that of moderate-intensity continuous exercise, and repeated acute high-intensity exercise will induce decreased myocardial contractility, increased markers of cardiac damage, and decreased ejection fraction^4^. Therefore, acute high-intensity exercise is not entirely beneficial to the myocardium. A healthy metabolic state will provide the myocardium with ATP to ensure the normal operation and improving the myocardial metabolism is critical to treat exercise-induced heart injury^5^.

Metabolomics is a new approach following genomics, proteomics, and transcriptomics, which has the advantages of high throughput, high specificity, and high sensitivity. Metabolomics can capture metabolic architecture under specific external stimuli and scan panoramic metabolites to reveal the transition between health and disease^6^. Nuclear magnetic resonance (NMR) spectroscopy and liquid chromatography-mass spectrometry (LC–MS) are the two most common technologies for the analysis of biological samples. While the application of metabolomics technology on sports remains unexplored.

Here, we focused on the metabolic signature of the myocardium during acute high-intensity exercise through combining animal and human experiments to explain the effect of acute high-intensity exercise on cardiac function from the perspective of material metabolism.

## Results

### Acute high-intensity exercise-induced myocardial injury

**Figure 1:**
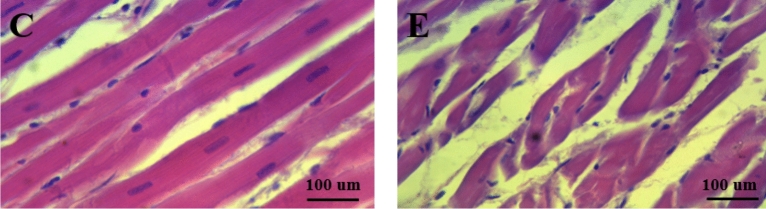
HE staining to observe myocardial morphology and structure.

Compared with group C, SOD activity, MDA level (*p* < 0.01), and cTnI content (*p* < 0.05) in group E increased significantly; GSH content (*p* < 0.01) in group E decreased significantly (Table 1). Furthermore, the myocardial fibers of rats in group C were dense and neatly arranged with no abnormal nuclear morphology and complete cell structure but the rat myocardium in group E occurred severe histopathological changes described as thin and disordered myocardial fibers, most of which were broken (Fig. 1).

**Table 1:** Serum oxidative stress and myocardial injury markers in rats.

	Group C	Group E
SOD	68.734 ± 7.773	89.892 ± 2.810**
MDA	3.694 ± 0.572	4.251 ± 0.383**
GSH	4.754 ± 0.683	3.214 ± 0.672**
cTnI	51.643 ± 0.816	60.419 ± 1.001*

**p < 0.01 versus C group, *p < 0.05 versus C group.

### QC analysis and Overall sample Hotelling’s T2

The UHPLC-Q-TOF MS ion chromatograms of the QC sample were overlapped well, indicating the instrument was in good condition and the experimental data was reliable (Supplementary Fig. S1). Overall sample Hotelling’s T2 analysis was used to detect outliers in this experiment. Here, all samples were within the 99% confidence interval and there were no outliers (Supplementary Fig. S2).

### Typical metabolic profile

**Figure 2:**
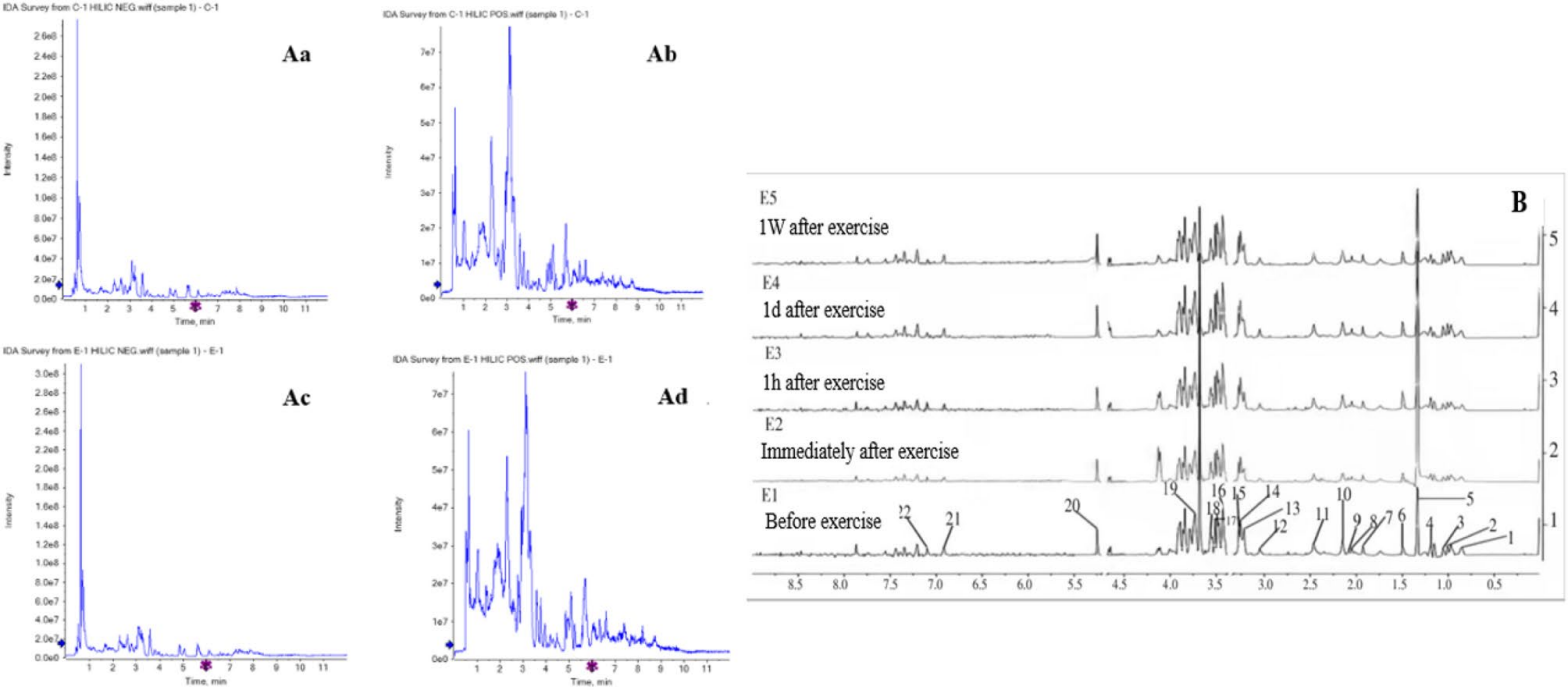
(**A**) The total ion current diagram of typical metabolites in a myocardial sample. (**a**) Group C negative ion current diagram, (**b**) Group C positive ion current diagram. (**c**) Group E negative ion current diagram. (**d**) Group E positive ion current diagram; (**B**) 1H-NMR spectrum of subject serum during different exercise periods.

Myocardial samples were detected by LC–MS to obtain typical metabolic spectrums (Fig. 2A). A total of 135 metabolites were identified in rat myocardial samples from group C and group E, and the identity, retention time (rt), and mass-to-charge ratio (m/z) of metabolites are shown in the Supplementary Table S2. The contours of the myocardial metabolites between the two groups had changed to different degrees, which can be found by further analysis. The ^1^H-NMR spectrum collected from subjects’ serum during the different periods of the exercise was shown in Fig. 2B, and we can see the differences in the peak intensities before and after acute high-intensity exercise. Consulted the Human Metabolome Database (HMDB) and related literature, 22 metabolic markers were identified between E1 and E2 (Supplementary Table S3).

### PLS-DA analysis results

**Figure 3:**
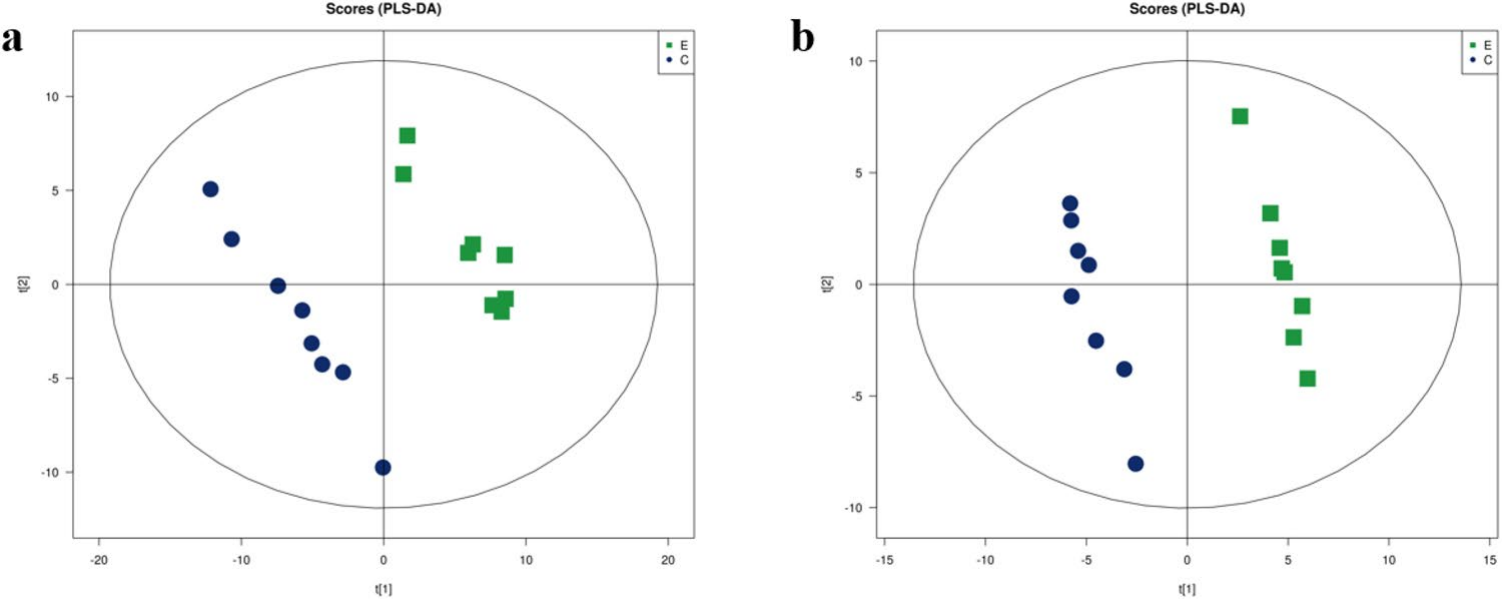
LC/MS based PLS-DA of rat myocardial samples from group C and group E. (**a**) Negative ions in group C/E, (**b**) positive ions in group C/E.

Partial least squares discrimination analysis (PLS-DA) was used to predict differences between two groups. This experiment supervised and identified all ion peaks obtained from the two groups after myocardial preconditioning and established a regression model. The model parameters (7 cycles of verification) showed the model establishment was stable and reliable (Supplementary Table S4). Constructed a PSL-DA model score plot (Fig. 3), the positive and negative ion points had a significantly separated trend between group C and group E, but had a concentrated trend within the group, indicating the metabolites of rat myocardium in two groups were different.

### OPLS-DA analysis results

**Figure 4:**
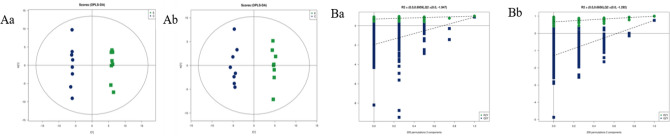
(**A**) LC/MS based OPLS-DA of rat myocardial samples from group C and group E. (**a**) Negative ions in C/E group, (**b**) positive ions in C/E group; (**B**) LC/MS based OPLS-DA permutation test diagram of rat myocardial samples from group C and group E. (**a**) Negative ions in group C/E, (**b**) positive ions in group C/E. Abscissa: permutation retention for permutation tests; Ordinate: the value of R2 or Q2.

Orthogonal PLS-DA (OPLS-DA) revises the experimental data from PLS-DA and enhances the significance of differences between groups. The model parameters were shown in Supplementary Table S5. In this study, R^2^_Y_ and Q^2^ were both greater than 0.5, indicating the two groups had significant differences and the OPLS-DA model was reliable. Constructed the OPLS-DA model score plot (Fig. 4A), we can see a clear separation between group C and group E and a clear concentration within the group, indicating the metabolites of rat myocardium before and after acute high-intensity exercise had significant differences.

The permutation test builds the OPLS-DA model 200 times by randomly changing the arrangement order of the categorical variable Y to obtain the R2 and Q^2^ values of the random model (Fig. 4B). On the same abscissa, the R^2^ value was greater than the Q^2^ value, which was well separated. The rightmost points of R^2^ and Q^2^ were both greater than the other points and the leftmost value of Q^2^ was less than 0, indicating the model verification had passed and the analysis of PLS-DA, OPLS-DA results was meaningful.

**Figure 5:**
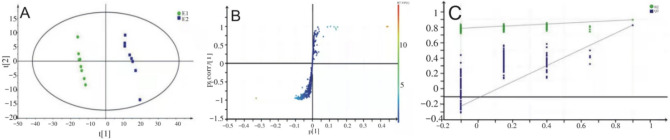
(**A**) ^1^H-NMR based OPLS-DA score plot of subject serum samples; (**B**) ^1^H-NMR based S-Plots of the OPLS-DA model from subject serum samples; (**C**) ^1^H-NMR based OPLS-DA permutation test diagram of subject serum samples.

Similarly, as can be seen from Fig. 5A, the subject serum sample in E1 and E2 was close, but the subjects’ serum sample between E1 and E2 was completely separated, indicating the metabolites of subjects’ serum changed significantly during acute high-intensity exercise. Variable importance for the projection (VIP) measures the impact strength and explanatory power of metabolite expression patterns on the classification and discrimination of each group sample, usually, VIP score > 1.0 is used as the screening standard for marker metabolites. Each point in Fig. 5B represents a variable, and the VIP value of points further from the origin is greater, which contributes more to the separation of subjects' serum samples. Figure 5C shows the verification of the OPLS-DA model holds.

### Univariate statistical analysis results

**Figure 6:**
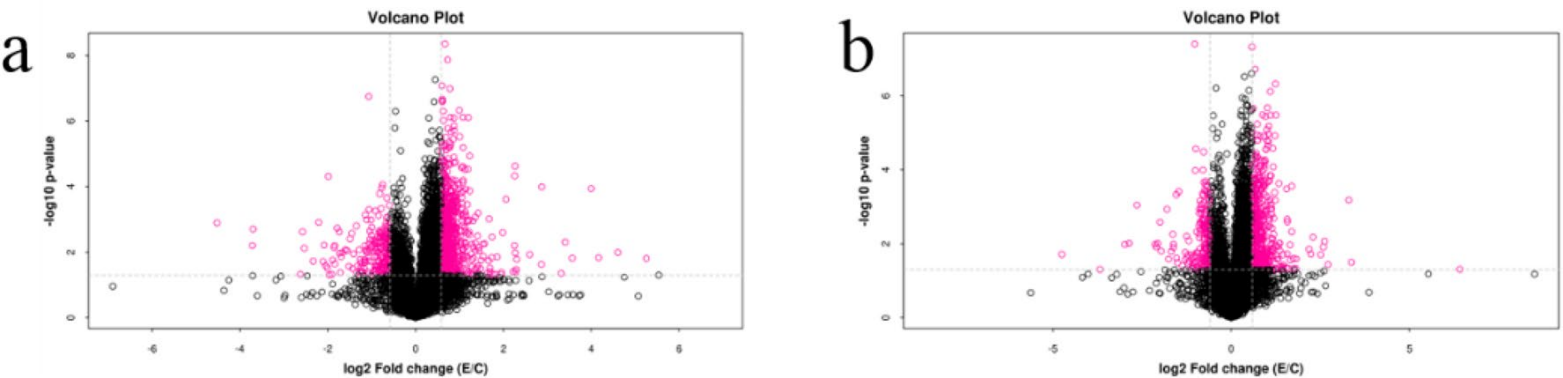
The volcano plot of rat myocardial samples. (**a**) Negative ions in group C/E; (**b**) positive ions in group C/E.

Combined *t*-test and FC analysis to make Volcano Plots of rat myocardial metabolites (Fig. 6). Visually displayed the significantly changed metabolites between the two groups and speed up the screening of potential metabolic markers involved in the pathway. Based on *p* < 0.05, FC > 1.5, or FC < 0.67, the substances represented by the red dots were the different metabolites of rat myocardium before and after acute high-intensity exercise.

### Comparison results of different metabolites

The VIP value obtained from OPLS-DA was used to screen the differential metabolites. The *t*-test and FC analysis judged the significance and changed trends of differential metabolites. VIP > 1.0, *p* < 0.05, FC > 1.5 represented the differential metabolites were significantly increased; VIP > 1.0, *p* < 0.05, FC < 0.67 represented the differential metabolites were significantly decreased. It was found there were 32 different metabolites in the rat myocardium sample between group C and group E (Table 2) and 14 different metabolites in subjects’ serum sample between E1 and E2 (Table 3). Furthermore, we observed before and after acute high-intensity exercise, the differential metabolites in human serum and rat myocardium were similar to a certain extent, which both referred to energy metabolism, amino acid metabolism, lipid metabolism, and glucose metabolism.

**Table 2:** Different metabolites and changed trend of rat myocardium in C/E group.

Mode	Quantity	Mass-to-charge ratio	Retention time (s)	Differential metabolites	Variation tendency
ESI +	1	120.079	150.693	Tyramine	↓**
ESI +	2	810.599	50.791	1-Stearoyl-2-oleoyl-sn-glycerol 3-phosphocholine (SOPC)	↑**
ESI +	3	496.336	216.480	1-Palmitoyl-sn-glycero-3-phosphocholine	↑**
ESI +	4	171.004	471.382	Glyceraldehyde 3-phosphate	↑*
ESI +	5	145.049	335.610	l-(−) Sorbose	↑*
ESI +	6	426.318	225.515	cholic acid	↓*
ESI +	7	123.054	456.870	Nicotinamide	↓*
ESI +	8	400.339	169.317	l-Palmitoylcarnitine	↑*
ESI +	9	134.044	449.359	l-Aspartate	↑*
ESI +	10	734.564	175.564	Phosphatidylcholine	↑*
ESI +	11	278.061	472.020	d-glucose 6-phosphate	↑*
ESI +	12	175.119	579.330	l-Arginine	↑*
ESI +	13	127.038	471.296	Larixinic Acid	↑*
ESI +	14	109.027	452.725	Quinone	↑*
ESI-	15	175.024	370.209	l-galacturonic acid	↑**
ESI-	16	267.195	68.536	Hexadecanedioic acid	↑**
ESI-	17	187.133	102.738	3-Hydroxycapric acid	↑**
ESI-	18	180.033	124.399	Acamprosate	↑**
ESI-	19	613.137	441.629	Cytidine monophosphate *N*-acetylneuraminic acid	↑**
ESI-	20	241.082	98.070	Thymidine	↑**
ESI-	21	103.039	188.660	d(−)-beta-hydroxy butyric acid	↑**
ESI-	22	125.035	73.774	Thymine	↑**
ESI-	23	111.020	85.016	Uracil	↑**
ESI-	24	295.226	62.648	l-Arabinono-1,4-lactone	↑**
ESI-	25	147.029	76.925	d-Arabinono-1,4-lactone	↑*
ESI-	26	191.016	99.294	d-Galactarate	↑*
ESI-	27	259.020	500.028	d-mannose 1-phosphate	↑*
ESI-	28	179.055	258.625	d-Mannose	↑*
ESI-	29	227.200	102.618	Myristic acid	↑*
ESI-	30	289.032	462.109	d-ribose 5-phosphate	↑*
ESI-	31	279.231	157.648	Linoleic acid	↑*
ESI-	32	303.231	137.760	Arachidonic acid (peroxide-free)	↑*

**p < 0.01 versus C group, *p < 0.05 versus C group; ↑ and ↓ maen increase and decrease of metabolite levels.

**Table 3:** Changes of serum metabolites in subjects before and after exercise.

δ (ppm)	Metabolite	E1 versus E2
0.86	Lipid	↓**
0.96	Leucine	↓**
1.01	Isoleucine	↓**
1.05	Valine	↓**
1.33	Lactate	↑**
2.08	Glutamate	↓**
2.14	Glutamine	↓**
3.05	Creatine	↓**
3.21	Carnitine	↓**
3.27	Betaine	↓**
3.49	Acetoacetic acid	↓**
3.57	Glycine	↓**
3.67	Glycerol	↓**
5.24	Glucose	↓**

**p < 0.01 versus C group, *p < 0.05 versus C group; ↑ and ↓ means increase and decrease of metabolite levels.

### Hierarchical clustering analysis of differential metabolites

**Figure 7:**
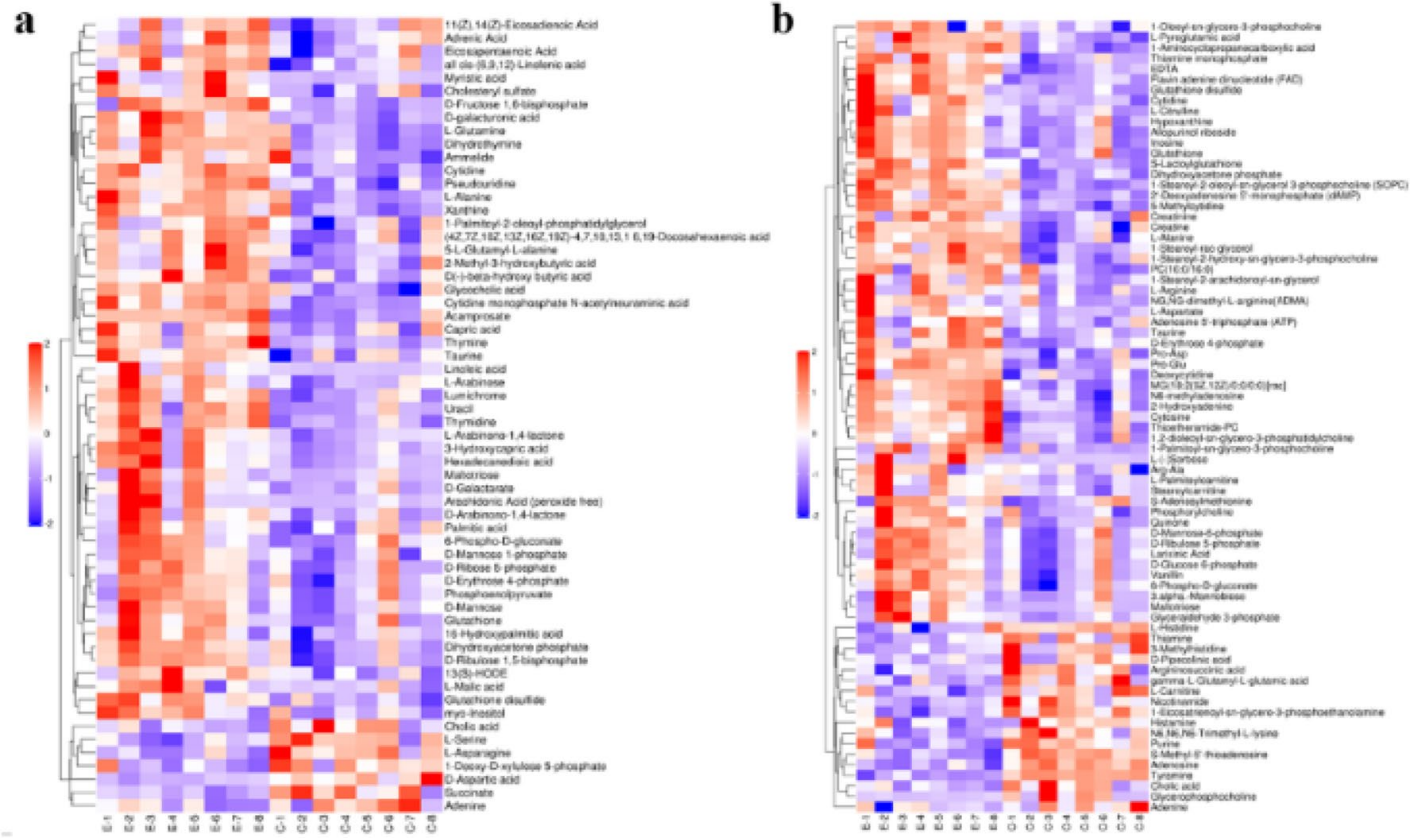
Hierarchical clustering of significantly differential Metabolites in rat myocardial samples. (**a**) Negative ions in group C/E; (**b**) positive ions in group C/E.

The differential metabolites of the myocardial samples in the two groups were handled by hierarchical cluster analysis (Fig. 7). The red represents significantly increased metabolites, and the blue represents significantly decreased metabolites. Here, the color in the same group was relatively concentrated but that of different groups was sharply contrasted, indicating the differences of myocardial metabolites within the group were small and the differences between the groups were obvious. The selected differential metabolites were reliable.

### Pathway analysis of target metabolites

**Figure 8:**
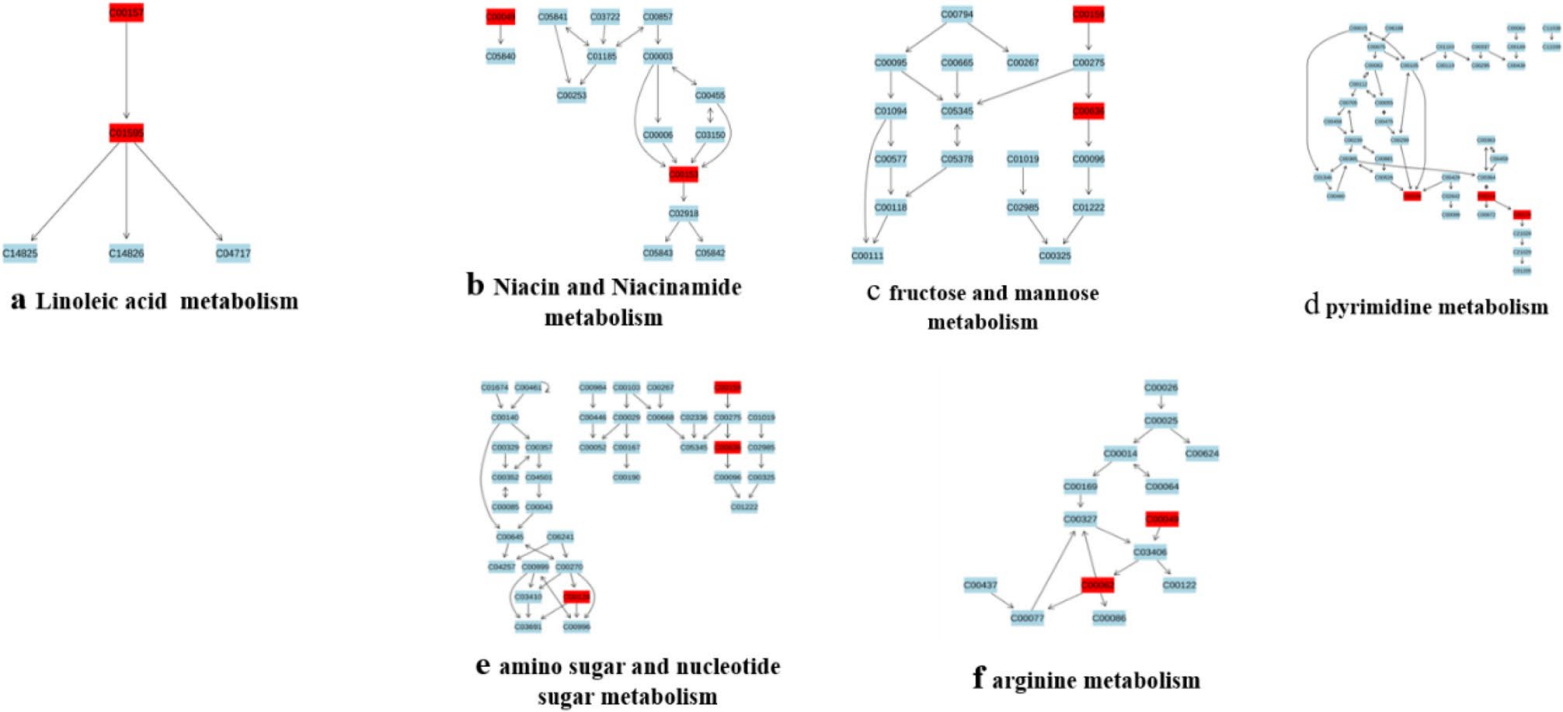
Target metabolic pathways involved in differential metabolites of rat myocardial samples. Red is the potential marker of the pathway involved in this study; Blue is not in the metabolites of this study.

MetaboAnalyst 4.0 was used to analyze the differential metabolites of rat myocardium in the two groups by the Met-PA approach. Imported the data of 32 different metabolites into Pathway Analysis to explore the weight of the metabolic pathways (Supplementary Fig. S3). There were 26 metabolic pathways involved during high-intensity exercise (Supplementary Table S6). Here, raw *p* < 0.05 and Pathway Impact > 0.05 were used as the critical point to screen the above-mentioned metabolic pathways. We found 6 potential target metabolic pathways that affected the myocardial metabolism of rats during acute high-intensity exercise, namely fructose and mannose metabolism, Linoleic acid metabolism, pyrimidine metabolism, nicotinate and nicotinamide metabolism, arginine metabolism, amino sugar and nucleotide sugar metabolism (Fig. 8).

### Metabolic markers of rat myocardium

The receiver operating characteristic curve (ROC) evaluated the diagnostic ability of differential metabolites during acute high-intensity exercise. Combining the area value (AUC) and *p*-value (*p* < 0.05) under the ROC curve, we found 12 potential metabolic markers included in the above 6 metabolic pathways, affecting the metabolism of acute high-intensity exercise. It was Thymine (AUC = 0.812), linoleic acid (AUC = 0.766), cytidine monophosphate N-acetylneuraminic acid (AUC = 0.859), l-Aspartate (AUC = 0.859), 1-Stearoyl-2-oleoyl-sn-glycerol 3-phosphocholine (AUC = 0.766), thymidine (AUC = 0.875), uracil (AUC = 0.75), d-mannose (AUC = 0.922), phosphorylcholine (AUC = 0.766), l-Arginine (AUC = 0.703), nicotinamide (AUC = 0.719), d-mannose 1-phosphate (AUC = 0.859) (Supplementary Table S7, Supplementary Fig. S4).

## Discussion

The regulatory outcomes and mechanisms of acute high-intensity exercise on cardiac function remain less clear. Acute high-intensity exercise is associated with cardiometabolic benefits, such as an increase of glutamine and arginine and a decrease of branched-chain amino acids and glutamic acid, which have a favorable effect on CVD progression^7^. Conversely, acute high-intensity exercise carries adverse risks as the higher the exercise intensity, the larger is the increase in myocardial oxygen demand, which may result in myocardial ischemia. Meanwhile, the increased oxygen metabolism will cause strong oxidative stress with a large accumulation of reactive oxygen species (ROS) and lactic acid, inducing myocardial structural damage^8^. Acute high intensity also promotes the release of inflammatory factors correlated with cardiac dysfunction. However, exercise-induced cardiac injury is usually transient, reversible, and has a favorable prognosis. Patients with exercise-related cardiac maladaptation have low mortality, no absolute exercise contraindications, and fine cardiac rehabilitation programs. Furthermore, patients who regularly engage in sports tend to recover better^1^.

This study used Metabolomics to explore the metabolic responses of the myocardium to acute high-intensity exercise and spotlight metabolic pathways and potential metabolic markers for future study. We found there were 6 target metabolic pathways during acute high-intensity exercise involved in 12 potential metabolic markers.

High-intensity exercise produces lots of ROS in a short time, which acts as a signal molecule to promote SOD activity^9^. Here, the serum SOD activity of rats in group E increased significantly, indicating the acute high-intensity exercise caused oxidative stress, therefore stimulating the enhancement of SOD activity. MDA is the main product of lipid peroxidation caused by ROS. Increased MDA is recognized as a symbol of oxidative stress^10^. Serum MDA declined both in moderate and small-intensity exercise. While, when the exercise intensity is greater than 70% VO_2max_, MDA will increase significantly^11^. In this study, the serum MDA of the rats in group E was significantly increased, suggesting exercised rats occurred strong oxidative stress again. Due to the increase of ROS and lipid peroxidation, GSH needs to be consumed to clear it. Therefore, oxidative stress usually happens accompanied by a decrease of GSH^12^. In our study, acute high-intensity exercise may lead to oxidative stress because of the GSH consumption of rat serum in group E. Serum cTnI, employed as an indicator of myocardial injury, will be released into the blood, when the structure and function of myocardial cells destroy^13^. Here, the serum cTnI of rats in group E was significantly increased so that we can infer acute high-intensity exercise caused myocardial injury combining HE staining results.

Phosphatidylcholine (PC), 1-Stearoyl-2-oleoyl-sn-glycerol 3-phosphocholine (SOPC), and linoleic acid (LA) participated in the linoleic acid metabolism pathway (Fig. 8a). After acute exercise, increased PC levels lead to impaired utilization of cardiac fatty acids and inflammation-mediated metabolic disorders, thus inducing heart failure, which was closely related to the conversion from PC to lysophosphatidylcholines (LPCs) under high oxidative stress conditions^14,15^. The biological effects of SOPC are consistent with those of PC. After excessive consumption of red meat, SOPC increased greatly and easily generated trimethylamine oxide (TMAO), which was a risk factor for the occurrence of CVD^16^. In our study, SOPC and PC of rat myocardium in group E increased significantly, suggesting acute high-intensity exercise induced cardiac oxidative stress, thereby possibly accelerating the production of harmful substances, such as LPCs and TMAO, which damaged myocardial health. Linoleic acid, a type of free fatty acid, is associated with early MI. Patients with diastolic dysfunction were found a significant increase of LA in the neointimal part of the myocardium, which improved lipid metabolism to provide energy for the heart^17^. LA also increased when the myocardium is in a pathological condition, inducing myocardial hypertrophy. Additionally, the oxidation products of LA will cause macrophage apoptosis and MI^18^. In this study, LA of rat myocardium in group E increased significantly, providing evidence that the utilization of myocardial fatty acids was up-regulated during acute high-intensity exercise. However, myocardial ischemia and oxidative stress are prone to generate oxidation products and disrupt lipid metabolism, causing heart damage.

d-mannose and d-mannose 1-phosphate participated in the fructose and mannose metabolism pathway (Fig. 8c). d-Mannose exists as a component of mannan, which is the raw material to form d-mannose-1-phosphate^19^. Due to structural similarity with glucose, d-mannose will snatch glucose transporter to produce d-mannose-6-Phosphoric acid, which disrupts the aerobic oxidation of glucose, accelerates glycolysis, and causes abnormal energy supply for the myocardium. Disorders of glucose metabolism will reduce angiogenesis and cardiac antioxidant capacity by affecting molecular signaling pathways, which ultimately lead to heart disease^20^. Studies showed ultramarathon runners had been detected high levels of d-mannose in their urine with the risk of myocardial ischemia^21^. d-mannose-1-phosphate easily reacts with proteins or lipids through the glycosylation process and generates glycosylation end products (AGEs) which easily cause myocardial lipid metabolism disorders, myocardial chronic inflammation, and cardiomyocyte apoptosis^19,22^. Myocardial pressure, corresponding to heart failure, is overloaded during high-intensity exercise, which will make material metabolism abnormal and produced more AGEs^23^. In this study, d-mannose and d-mannose 1-phosphate of rat myocardium in group E increased significantly, which made us wonder the harmful effect of acute high-intensity exercise might act on glucose metabolism, such as enhanced glycolysis and production of AGEs, inducing myocardial injuries.

Cytidine-monophosphate *N*-acetylneuraminic acid (CMP-Neu5Ac), d-mannose 1-phosphate, and d-mannose participated in amino sugar and nucleotide sugar metabolism pathway (Fig. 8e). CMP-Neu5Ac is the activated form of Neu5Ac and the two are positively correlated^24^. An increase of CMP-Neu5Ac caused cardiomyocyte apoptosis and inflammatory response, interfering with lipid metabolism and causing myocardial injuries^25^. Besides, Neu5Ac increases the permeability of endothelial cells and promotes the release of oxidized low-density lipoproteins, which will destroy the intravascular microenvironment and cause CVD^26^. Thus, Neu5AC concentration can be served as a marker of heart disease. Here, CMP-Neu5Ac of rat myocardium increased significantly after acute high-intensity exercise, which might be one of the reasons why exercise caused heart injuries.

Nicotinamide (NAM) and l-Aspartate (Asp) participated in nicotinate and niacinamide metabolism pathway (Fig. 8b). NAM is essential for cardiovascular health through enhancing myocardial homeostasis and inhibiting cardiomyocyte apoptosis^27^. Excessive exercise is attributed to myocardial ischemia and hypoxia, leading to significantly decreased NAM and exercise-induced fatigue in rats^28,29^. In our study, significantly decreased NAM during acute high-intensity exercise was found, suggesting reduced exercise-induced NAM of myocardium might induce cardiac pathological damage. Asp is an important substrate of gluconeogenesis with the effects of protecting cardiovascular health and promoting fatigue recovery^30^. Supplementing Asp during high-intensity exercise can promote gluconeogenesis, reduce the accumulation of lactic acid and maintain normal blood PH value^31,32^, not only to alleviate exercise fatigue but also to exert a beneficial effect on myocardial metabolism. Here, Asp of rat myocardium in group E increased significantly, indicating exercise-induced changes in Asp content may have great benefit for cardiac health by resisting oxidative stress and improving myocardial metabolism.

l-arginine (l-Arg) and Asp participated in the arginine metabolism pathway (Fig. 8f). l-Arg plays a positive regulatory role in the bioavailability of nitric oxide, which is beneficial for improving coronary perfusion and cardio-pulmonary function^33^. During strenuous exercise, l-Arg up-regulated the utilization of glucose and lipid through PI3K/Akt signal pathway and mediate myocardial oxidative stress by cleaning ROS and enhancing antioxidant enzymes activity^34,35^. In our study, l-Arg and Asp in rat heart increased significantly after acute high-intensity exercise, which performs favorable functions on myocardial response to exercise, protecting the heart against exercise-induced injuries.

Uracil, thymidine, and thymine participated in the pyrimidine metabolism pathway (Fig. 8d). Uracil in mice myocardium increased significantly during the recovery period of high-intensity running, which was essential for myocardial repair by maintaining cardiac output and reducing inflammation-mediated myocardial apoptosis^36^. The plasma thymine in mice with acute myocardial ischemia increased significantly, which was closely related to heart disease^37^. Moreover, a significant decrease of thymidine in rats serum represents a biomarker of early acute MI^38^. After strenuous exercise, increased thymine in rats’ serum had been confirmed which will induce cardiac ischemia. Thymidine supplementation can promote Cardiomyocyte regeneration and provide energy for the myocardium^39^. Here, thymine in group E increased significantly but uracil and thymidine increased significantly, suggesting myocardium might have pathological changes such as ischemia and hypoxia during acute high-intensity exercise, which will activate cardiac defense by regulating pyrimidine metabolism.

To explore myocardium metabolic response to exercise in further depth, we detected metabolic alteration in human blood after acute high-intensity exercise. Glucose (Glu) and lactic acid (La) are energy supply substances and products of the glycolysis system. Due to myocardial hypoxia and ischemia, the aerobic oxidation of glucose decreases, which promotes the conversion of pyruvate to La to resist energy deficiency^40^. We found serum Glu decreased but La increased after acute high-intensity exercise, indicating myocardium might be in ischemia and hypoxia conditions because of the decrease of glucose aerobic oxidation and the increase of glycolysis to maintain ATP levels. Increased serum glycerol (TG) is an indicator of fine lipolysis, which increased less or even decreased in subjects with exercise-induced myocardial ischemia^41^. Here, acute high-intensity exercise reduced serum lipid and TG, which suggested acute high-intensity exercise up-regulated lipid catabolism but impaired utilization of fatty acids, possibly resulting in myocardial ischemic. Acetoacetate is a kind of carcass (KB). During distance cycling, the KB of athletes was reduced by oxidation and increased significantly during the exercise recovery period^42^. The decrease of KB in our study indicated acute high-intensity exercise enhanced energy supply from KB disintegration, reducing serum KB concentration in response to exercise-induced glycogen depletion. During acute exercise, glutamate (Glu) was utilized and glutamine (Gln) was released to promote ammonia metabolism^43^. In our study, Glu and Gln decreased significantly, indicating exercise increased blood ammonia, promoted Glu absorption and Gln release to neutralize toxic effects of blood ammonia. However, due to excessive intensity, Gln was decomposed to provide energy. Glycine (Gly) is the raw material for the synthesis of glutathione and creatine (Cr)^44^. We observed a decrease of serum Gly and Cr immediately after exercise because Gly was used to synthesize Cr which was further utilized to improve ATP resynthesis. Branched-chain amino acids (BCAAs) include leucine (Leu), isoleucine (Ile), and valine (Val), which replace glycolysis under anaerobic conditions to supply energy and reduce during acute exercise to resist the occurrence of CVD^45^. Here, BCAAs in subject serum decreased after acute high-intensity exercise, and we speculated BCAAs were consumed as energy substrates to improve cardiac metabolism. Carnitine (CAR) is an activated fatty acid carrier on the mitochondrial membrane, carrying fatty acids into mitochondria for β-oxidation^46^, while betaine (Bet) provides methyl groups for CAR synthesis^47^. In our study, CAR and Bet decreased significantly after acute high-intensity exercise, suggesting lipolysis utilizes betaine for energy, and Bet was also consumed as a methyl donor. Similar to animal experiments results, after acute high-intensity exercise, the human body protects against exercise stimulation and maintains myocardial homeostasis by regulating the metabolic pathways of lipids, carbohydrates, and amino acids.

## Conclusion

Combining animal and human experiments, this study found dramatic changes in myocardial metabolites and the major altered metabolic pathway due to acute high-intensity exercise, which provided insights that exercise-induced cardiac dysfunction may be related to lipid peroxidation, anaerobic glycolysis, and energy metabolism caused by oxidative stress and the favorable shift of amino acid and nucleotide metabolism can repair damaged myocardium and provide energy for myocardium to maintain physiological heart function. Taken together, acute high-intensity exercise may induce cardiac diseases, which will turn on the "beneficial switch" of the body's defense mechanism to attenuate exercise-induced myocardial adverse changes. There are still limitations in our study. Echocardiographic testing should be performed to observe changes in cardiac structure and function during exercise in real-time and different recovery stages after exercise and cardiometabolic changes in patients with CVD should also be detected to more comprehensively explore the myocardial metabolic response to acute high-intensity exercise.

## Materials and methods

### Ethics, consent, and permissions

All animal studies were approved by the Ethics Committee of Scientific Research in Shanxi University, which conformed to local and international guidelines on the ethical use of animals and complied with the ARRIVE guidelines; The human study was approved by the Ethics Committee of Shanxi University (No. SXULL2020064), which followed the declaration of Helsinki, and signed informed consent was obtained from all subjects before the experiment.

### Animal and human study

Sixteen male Sprague–Dawley rats (Vital river laboratory animal technology biotech, Beijing, China) weighing 190–200 g were randomly divided into a control group (C) and an acute high-intensity exercise group (E), 8 in each group. Group C was raised quietly and normally; Group E received an acute high-intensity exercise intervention. Raising condition: Light and dark cycled every 12 h, free access to tap water and food, the temperature was 26–28 °C, the humidity was 50–60%.

8 males from Physical Education Institute were selected as subjects whose basic information was shown in Supplementary Table S1. They were all healthy without bad living habits and had undergone routine physical examinations, excluding the history of major diseases, upper respiratory tract infection, and cardiovascular disease. Subjects volunteered to follow the requirements of the experiment strictly.

After exercise (within 45 s), each rat was decapitated immediately. Rat myocardial samples were stripped, snap-frozen in liquid nitrogen after washing with ice physiological saline, and stored at − 80 °C; Rats blood samples were collected in sterile vacutainers, then stored at 4 °C to prevent sample degradation. Subjects underwent brachial vein blood collection without eating anything in the morning, marked as E1; immediately after exercise (within 45 s), the second blood collection was performed, marked as E2; 5 ml blood was collected in sterile vacutainers each time, and also stored at 4 °C. After centrifugation at 3000 rpm and 4 °C for 20 min, Subjects and rats serum samples were all placed in clean EP tubes and stored at − 80 °C until analysis.

### Exercise program

Rats exercised on a three-track rat-special treadmill. On the first day, group E performed an adaptive exercise, 3 m/min, 5 min, 1 week in total; On the 8th day, Exercise intensity pre-adaptation was conducted with the initial speed of 3 m/min, 3 min, separated by 1 min of rest. The speed increased by 3 m/min after rest until the rat was exhausted (rats stayed at the end of the treadmill, run on the abdomen after an electric shock, and returned to the original place within three seconds), repeated 5 times^48^; On the 20th day, the exercise capacity was tested, which based on the Bedford’s article^49^, the average exhaustion speed was 21 m/min (75% VO2max); On the 28th day, did the final acute high-intensity treadmill exercise, 24 m/min, − 16° inclination. The exercise time was 5 min with 1 min rest, cycling 8 times.

Subjects pedaled the power bicycle with full force for 30 s × 3 (load was 0.075 kg/kg BW), rested for 3 min after completion, and cycled 3 times^50^.

### Reagents and instruments

Acetonitrile; Ammonium acetate; Ammonia; MDA kit, SOD kit, GSH kit, cTnI kit. Triple TOF 6600+ mass spectrometer; 1290 Infinity LC ultra-high pressure liquid chromatography; ACQUITY UPLC BEH Amide column; Bruker AVANCE III 600 MHz NMR spectrometer; 5430R low-temperature high speed centrifuge.

### Detection of MDA, SOD, GSH, and cTnI

Rat serum superoxide dismutase (SOD), malondialdehyde (MDA), glutathione (GSH), creatine kinase (cTnI) was detected by spectrophotometer (UV − 6100 s, Mapada, Shanghai, China) according to the kit’s instructions (Callegari, Italy).

### Histologic analysis

Fixed myocardium tissues in 10% neutral buffered formalin, stained sections with standard hematoxylin–eosin (H&E) and observed structural changes of myocardial tissue under a microscope.

### Sample pretreatment

For LC/MS analysis, Mixed 80 mg of rat myocardium with 200 µl water in a 2 ml EP tube, vortexed for 1 min, and put the pre-cooled 800ul acetonitrile/methanol mixture (1:1, V/V) into the EP tube. Took the solution into the refrigerator for 1 h to remove the sample protein after two low-temperature ultrasonic treatments (30 min/time) then centrifugated at 4 °C and 14000 rpm for 20 min. The supernatant was vacuum freeze-dried and stored at − 80 °C. Added 100 µl acetonitrile/water (1:1/V:V) mixture and blended thoroughly to reconstitute sample. After centrifugation at 4 °C and 14000 rpm for 15 min, took the supernatant for mass spectrometry injection analysis. The myocardium sample was prepared as quality control samples (QC) for testing the instrument and system status.

For NMR analysis, Mixed 450ul of subject serum with 900uL methanol in a 2 mL EP tube, vortexed for 2 min to react fully then centrifuged at 4 °C and 13000 rpm for 30 min. Took 900ul supernatant into 5 ml EP tube, dried by a nitrogen blower with an air source, and added 600uL of PBS buffer to reconstitute. After fully dissolving, added 550 µL to 5 mm NMR tube for NMR spectroscopy analysis.

### Chromatographic conditions

Put the myocardial sample into the autosampler and added the HILIC chromatographic column. The injection volume was 2ul, the column temperature was 25 °C and the flow rate was 0.3 ml/min. Phase A: water + 25 mmol/L ammonium acetate + 25 mmol/L ammonia; Phase B: acetonitrile. Gradient elution procedure: 0–0.5 min, 95%B; 0.5–7 min, 95–65%B; 7–8 min, 65–40%B; 8 ~ 9 min, 40%B; 9–9.1 min, 40–95%B; 9.1–12 min, 95%B. QC was randomly added to reduce the signal fluctuations.

### Mass spectrometry conditions

Electrospray ionization source setting parameters:ion source gas 1:60; ion source gas 2:60; curtain gas: 30; source temperature: 600 °C; ions apart voltage floating: ± 5500 V. The secondary mass spectrum was obtained through data correlation acquisition in high-sensitivity mode. Related setting parameters: collision energy: 35 ± 15 eV; declustering potential: ± 60 V; candidate ions to monitor per cycle: 6; exclude isotopes: 4 Da.

### LC/MS data processing

The raw data were converted to mzXML format by ProteoWizard, and then the XCMS program was used for peak alignment, retention time correction, and extraction of peak areas. Metabolite structures were identified by accurate mass matching (< 25 ppm) and headphone spectrum matching. The laboratory was searched to build a database. After the data was preprocessed by Pareto-scaling, multivariate data analysis was performed.

### NMR sequence setup

subject serum sample processed via ^1^H-NMR spectroscopy was detected using a Bruker AVANCE III 600 MHz NMR spectrometer, where scanning could be carried out up to 64-fold with the Bruker 5 mm BBO probe at a 600.13 MHz proton frequency, a 298 K acquisition temperature, a Carr–Purcell–Meiboom–Gill pulse sequence, a spin relaxation of delay of 320 ms and a spectral width of 8kHZ with 64 k data points of free induction decay.

### NMR data processing

Processed subject blood maps using MestreNova software to perform Fourier transform, adjusted the baseline, and corrected the phase. The chemical shift of total suspended particulate was calibrated as δ0.00. The spectrum from 0.5 to 9 ppm was divided into equal widths after manually removing the water peak (δ4.5–6.2), and the integral width was 0.01 ppm.

### Statistical analysis

Data collected from LC–MS and NMR were saved in Excel to normalize for multivariate statistical analysis. Used SPSS 24.0 for univariate statistical analysis including Student’s t-test and Fold change analysis (FC analysis). SIMCA 14.1 was used for supervised statistical analysis. Statistically significant compounds were evaluated by using ROC curve analysis. Made the volcano map and cluster map. Searched Met-PA database and Kyoto Encyclopedia of Genes and Genomes (KEGG) pathway database^51,52^ (http://www.genome.jp/kegg/pathway.html) to screen out target metabolic pathways and potential metabolic markers.

## Supplementary Information

Supplementary Information.
