# Supplementary material for: Effect of acute high-intensity exercise on myocardium metabolic profiles in rat and human study via metabolomics approach
Source: Sci Rep. 2022 Apr 26;12:6791. doi: 10.1038/s41598-022-10976-5 (PMC9042871; doi:10.1038/s41598-022-10976-5)
Supplement: Supplementary file 1 — Supplementary Information. [file 41598_2022_10976_MOESM1_ESM.pdf]

Supplementary information

Effect of Acute High-Intensity Exercise on Myocardium Metabolic Profiles in Rat and Human Study via Metabolomics Approach

Lijun Wu[1], Jiayi Wang[1]*, Xiuhui Cao[1], Yue Tian[1], Jia Li[1]

1 School of Physical Education, Shanxi University, Taiyuan, Shanxi, China.
* Corresponding author: Jiayi Wang, School of Physical Education, Shanxi University, NO. 92, Wucheng Road, Taiyuan, Shanxi, China, 030006. Email: 2458349847@qq.com.

Table of contents

Supplementary Tables

Page	Supplementary Table	Description
2	S1	Basic information of participants involved in this study.
2	S2	Summary of LC/MS analysis identified metabolites in rat myocardial samples from group C and Group E.
5	S3	Summary of ^{1}H-NMR analysis identified metabolites in subject serum samples from group E1 and group E2.
6	S4	PLS-DA analysis model parameter table
6	S5	OPLS-DA analysis model parameter table
6	S6	Differential metabolite pathway analysis results obtained through Met-PA
8	S7	KEGG ID of potential metabolic markers (http://www.genome.jp/kegg/compound/) [52] [53]

Supplementary Figures

Page	Supplementary Figure	Description
9	S1	Overlapping TIC spectra of negative and positive ion modes of QC samples
9	S2	Hotelling's T2 diagram in negative and positive ion mode of the sample
9	S3	Acute high-intensity exercise metabolic pathways constructed by Met-PA database
10	S4	Assessment of the diagnostic power of receiver operating characteristic curves for the differential metabolites acute high-intensity exercise.

Table S1

Basic information of participants involved in this study.

	Age	Height（m）	Weight（kg）	BMI	100m result（s）
Subjects	19.06±0.60	1.76±0.03	69.30±1.50	21.89±0.97	12.6±0.2

Table S2

Summary of LC/MS analysis identified metabolites in rat myocardial samples from group C and Group E.

A. Positive ion mode

Name	Metabolite	rt (s)	m/z
M120T151	Tyramine	150.693	120.079
M258T224	5-Methylcytidine	224.143	258.107
M293T431_2	EDTA	430.529	293.096
M250T94	Adenosine	93.599	250.092
M337T195_2	MG (18:2(9Z,12Z)/0:0/0:0) [rac]	194.661	337.272
M298T95	S-Methyl-5'-thioadenosine	94.521	298.096
M245T412	Pro-Glu	411.7275	245.113
M380T390	S-Lactoylglutathione	389.698	380.110
M332T415	2'-Deoxyadenosine 5'monoph-osphate (dAMP)	414.587	332.073
M786T396	Flavin adenine dinucleotide (FAD)	396.179	786.160
M811T51	1-Stearoyl-2-oleoyl-sn-glycerol 3-phosphocholine (SOPC)	50.791	810.599
M112T205_2	Cytosine	204.739	112.050
M231T423	Pro-Asp	422.550	231.097
M282T275	N6-methyladenosine	274.921	282.119
M84T367_2	1-Aminocyclopropanecarboxyl-ic acid	367.269	84.044
M152T227	2-Hydroxyadenine	227.419	152.055
M504T193	1-Eicosatrienoyl-sn-glycero-3-phosphoethanolamine	193.391	504.302
M231T435	Dihydroxyacetone phosphate	435.3	231.025
M613T492	Glutathione disulfide	492.361	613.158
M537T215	Allopurinol riboside	214.962	537.167
M228T205	Deoxycytidine	204.742	228.097
M269T215	Inosine	214.962	269.087
M147T367_2	L-Pyroglutamic acid	367.150	147.076
M201T391	D-Erythrose 4-phosphate	391.191	201.014
M126T291_2	Taurine	291.211	126.022
M345T485	Thiamine monophosphate	485.36	345.077
M90T343_2	L-Alanine	342.775	90.054
M524T186_18	1-Stearoyl-2-hydroxy-sn-glycero-3-phosphocholine	185.966	524.369
M244T237	Cytidine	237.101	244.093
M496T216	1-Palmitoyl-sn-glycero-3-phosphocholine	216.48	496.336
M759T124	Thioetheramide-PC	124.188	758.568
M341T154	1-Stearoyl-rac-glycerol	153.975	341.303
M121T111	Purine	111.126	121.049
M156T485	L-Histidine	484.903	156.075
M132T343_3	Creatine	342.775	132.076

M787T141	1,2-dioleoyl-sn-glycero-3-phosphatidylcholine	140.515	786.598
M277T459	gamma-L-Glutamyl-L-glutamic acid	459.410	277.102
M184T468_5	Phosphorylcholine	468.045	184.073
M162T409	L-Carnitine	409.250	163.111
M176T385	L-Citrulline	385.268	176.102
M123T457	Nicotinamide	456.87	123.054
M400T169	L-Palmitoylcarnitine	169.317	400.340
M171T471	Glyceraldehyde 3-phosphate	471.382	171.004
M189T515	N6, N6, N6-Trimethyl-L-lysine	514.805	189.159
M265T387	Thiamine	387.128	265.113
M170T374	3-Methylhistidine	373.66	170.091
M134T449	L-Aspartate	449.359	134.044
M213T441	D-Ribulose 5-phosphate	441.475	213.014
M114T167_2	Creatinine	166.775	114.065
M219T461	Vanillin	460.988	219.004
M137T215_5	Hypoxanthine	215.155	137.046
M291T465	Argininosuccinic acid	465.233	291.129
M735T176	PC (16:0/16:0)	175.564	734.564
M426T226	Cholic acid	225.515	426.318
M278T472	D-Glucose 6-phosphate	472.02	278.061
M259T415	6-Phospho-D-gluconate	415.291	259.019
M628T191	1-Stearoyl-2-arachidonoyl-sn-glycerol	190.834	627.530
M308T399	Glutathione	399.393	308.089
M175T579	L-Arginine	579.330	175.119
M127T471	Larixinic Acid	471.296	127.038
M109T453	Quinone	452.725	109.027
M203T483_2	NG, NG-dimethyl-L-arginine (ADMA)	482.956	203.150
M145T336_2	L- (-) Sorbose	335.610	145.049
M508T484	Adenosine 5'-triphosphate (ATP)	483.769	508
M258T473	Glycerophosphocholine	473.364	258.108
M428T166	Stearoylcarnitine	166.13	428.371
M522T187_2	1-Oleoyl-sn-glycero-3-phosphocholine	187.149	522.352
M522T447	Maltotriose	447.474	522.2
M147T552	D-Pipecolinic acid	551.623	147.112
M261T472	D-Mannose-6-phosphate	471.89	261.036
M112T366	Histamine	366.495	112.086
M136T209	Adenine	208.667	136.06
M245T334_5	Arg-Ala	334.247	245.150
M360T388	3.alpha. - Mannobiose	387.904	360.147
M399T512	S-Adenosylmethionine	512.254	399.141

B. Negative ion mode

Name	Metabolite	rt (s)	m/z
M180T124	Acamprosate	124.399	180.033
M242T237	Cytidine	237.006	242.078
M613T442	Cytidine monophosphate-N-acetylneuraminic acid	441.629	613.137
M175T370	D-galacturonic acid	370.209	175.024
M243T240	Pseudouridine	240.384	243.062
M145T368_2	L-Glutamine	367.600	145.062

M127T368_2	Dihydrothymine	367.610	127.051
M241T98	Thymidine	98.07	241.082
M611T492	Glutathione disulfide	492.026	611.143
M88T342_2	L-Alanine	341.941	88.04
M464T183	Glycocholic acid	183.455	464.312
M241T61	Lumichrome	60.688	241.073
M339T431	D-Fructose-1,6-bisphosphate	431.26	338.986
M103T189	D (-)-beta-hydroxy butyric acid	188.661	103.039
M125T74	Thymine	73.773	125.035
M369T436	D-Ribulose 1,5-bisphosphate	436.2	368.998
M131T373	L-Asparagine	372.781	131.045
M151T217_2	Xanthine	216.596	151.026
M229T437	Dihydroxyacetone phosphate	436.812	229.012
M117T389	Succinate	389.387	117.019
M111T85_2	Uracil	85.016	111.02
M104T372	L-Serine	372.413	104.034
M267T69	Hexadecanedioic acid	68.536	267.195
M187T103_2	3-Hydroxycapric acid	102.738	187.133
M295T63	L-Arabinono-1,4-lactone	62.648	295.226
M199T340	5-L-Glutamyl-L-alanine	339.909	199.069
M132T450	D-Aspartic acid	450.009	132.029
M277T45	All cis-(6,9,12)-Linolenic acid	45.042	277.216
M327T138	(4Z,7Z,10Z,13Z,16Z,19Z)-4,7,10,13,1-6,19-Docosahexaenoic acid	138.116	327.231
M295T43	13(S)-HODE	43.115	295.226
M117T149	2-Methyl-3-hydroxybutyric acid	148.583	117.055
M147T77	D-Arabinono-1,4-lactone	76.925	147.029
M407T225	Cholic acid	224.977	407.277
M149T152_2	L-Arabinose	151.749	149.045
M213T347	1-Deoxy-D-xylulose-5-phosphate	347.271	213.016
M191T99	D-Galactarate	99.294	191.016
M301T43	Eicosapentaenoic Acid	43.083	301.215
M465T25_2	Cholesteryl sulfate	25.455	465.303
M124T291_2	Taurine	290.958	124.007
M306T402	Glutathione	401.973	306.074
M259T500	D-Mannose 1-phosphate	500.028	259.021
M271T44	16-Hydroxypalmitic acid	44.396	271.226
M179T259_2	D-Mannose	258.625	179.055
M227T103_3	Myristic acid	102.618	227.2
M289T462	D-Ribose 5-phosphate	462.109	289.032
M179T388_2	myo-Inositol	387.861	179.056
M128T368_2	Ammelide	367.607	128.035
M748T72	1-Palmitoyl-2-oleoyl-phosphatidylglycerol	71.804	747.517
M279T158	Linoleic acid	157.648	279.231
M303T138	Arachidonic Acid (Peroxide free)	137.76	303.231
M134T206	Adenine	206.21	134.046
M171T53_2	Capric acid	52.625	171.138
M503T447	Maltotriose	447.351	503.158
M255T46_1	Palmitic acid	45.689	255.233

M307T42	11(Z),14(Z)-Eicosadienoic Acid	41.807	307.262
M331T39	Adrenic Acid	39.015	331.262
M133T406_2	L-Malic acid	406.150	133.014
M257T394	6-Phospho-D-gluconate	393.683	257.005
M199T449	D-Erythrose 4-phosphate	449.261	199
M227T412	Phosphoenolpyruvate	411.837	226.996

Table S3

Summary of ^{1}H-NMR analysis identified metabolites in subject serum samples from group E1 and group E2.

**Number**	**Metabolite**	**δ（^{1}H shift (ppm)/Multiplicity/Coupling constant(HZ)）**
1	Lipid	0.84(m)
2	Isoleucine	0.94(t, 7.4)<br>1.01(d, 7.0)
3	Leucine	0.96(d)<br>3.73(m)
4	Valine	0.99(d, 7.1)<br>1.05(d, 7.0)
5	Lactate	1.33(d, 6.9)<br>4.12(q, 6.9)
6	Alanine	1.48(d, 7.3)<br>3.77(m)
7	Acetate	1.93(s)
8	N-acetyl-glycoprotein	2.04(s)
9	Glutamate	2.08(m)
10	Glutamine	2.14(m)<br>2.45(m)
11	Citrate	2.54(d, 16.1)<br>2.65(d, 16.2)
12	Creatine	3.05(s)
13	Carnitine	3.21(s)
14	Choline Phosphate	3.22(s)
15	Trimetlylamine oxide	3.26(m)

16	Betaine	3.27(m)<br>3.89(s)
17	Acetoacetic acid	3.49(s)
18	Glycine	3.57(s)
19	Glycerol	3.67(m)<br>3.78(m)
20	Glucose	5.24(d, 3.7)<br>4.64(d, 8.0)
21	Tyrosine	6.90(m)<br>7.19(m)
22	Histidine	7.10(s)

s: singlet; d: doublet; t: triplet; q: quartet; m: multiplet; dd: doublet of doublet.

Table S4

PLS-DA analysis model parameter table

**Grouping**	**Negative ion mode**				**Positive ion mode**			
	A	R^2X(cum)	R^2Y(cum)	Q^2(cum)	A	R^2X(cum)	R^2Y(cum)	Q^2(cum)
**E/C**	3	0.477	0.999	0.897	2	0.242	0.995	0.754

A: the number of main components; R^2: the explanatory rate of model variables to X or Y; Q^2: the predictive ability of the model; the closer R^2Y and Q^2 are to 1, the more stable and reliable the model is; the model is stable and reliable when Q^2 is greater than 0.5

Table S5

OPLS-DA analysis model parameter table

**Grouping**	**Negative ion mode**				**Positive ion mode**			
	A	R^2X(cum)	R^2Y(cum)	Q^2(cum)	A	R^2X(cum)	R^2Y(cum)	Q^2(cum)
E/C	1+2	0.477	0.999	0.817	1+1	0.242	0.995	0.796

A: the number of main components; R^2: the explanatory rate of model variables to X or Y; Q^2: the predictive ability of the model; the closer R^2Y and Q^2 are to 1, the more stable and reliable the model is; the model is stable and reliable when Q^2 is greater than 0.5

Table S6

Differential metabolite pathway analysis results obtained from rat myocardial samples through Met-PA

Pathway	Total	Expected	Hits	Raw p	FDR	Impact
Linoleic acid metabolism	5	0.08284	2	0.00282	0.2148	1
Arginine metabolism	14	0.23194	2	0.01365	0.39832	0.07614
Amino sugar and nucleotide sugar metabolism	37	0.61299	3	0.02228	0.39832	0.07043
Niacin and Niacinamide metabolism	15	0.24851	2	0.02473	0.39832	0.1943
Pyrimidine metabolism	39	0.64612	3	0.02963	0.39832	0.17143
Biosynthesis of neomycin, kanamycin and gentamicin	2	0.03314	1	0.02963	0.39832	0
Fructose and mannose metabolism	18	0.29821	2	0.03329	0.39832	0.12422
Biosynthesis of Pantothenic Acid and CoA	19	0.31478	2	0.03937	0.39832	0
Beta-alanine metabolism	21	0.34791	2	0.04664	0.42614	0
Biosynthesis of unsaturated fatty acids	36	0.59642	2	0.1179	0.82529	0
Glycerophospholipid metabolism	36	0.59642	2	0.1179	0.82529	0.11182
Arachidonic acid metabolism	36	0.59642	2	0.1179	0.82529	0.33292
Aminoacyl-tRNA biosynthesis	48	0.79523	2	0.18753	1	0
Alpha-linolenic acid metabolism	13	0.21537	1	0.19592	1	0
Histidine metabolism	16	0.26508	1	0.23559	1	0
Starch and sucrose metabolism	18	0.29821	1	0.26098	1	0.13851
Pentose phosphate pathway	21	0.34791	1	0.29756	1	0.18501
Galactose metabolism	27	0.44732	1	0.36557	1	0
Alanine aspartate and glutamic acid	28	0.46388	1	0.37627	1	0.22356
Inositol phosphate metabolism	30	0.49702	1	0.39716	1	0
Arginine and proline metabolism	38	0.62956	1	0.47419	1	0.05786
Biosynthesis of primary bile acids	46	0.76209	1	0.54172	1	0
Metabolic degradation of fatty	39	0.64612	1	0.48312	1	0
Tyrosine metabolism	42	0.69583	1	0.50907	1	0.02463
Biosynthesis of fatty acids	47	0.77866	1	0.54955	1	0
Purine metabolism	66	1.09342	1	0.67608	1	0.01334

Total: the total number of compounds in the pathway; Expected: the expected value; Hits: the number of accurate matches in the uploaded marker data; Raw P: the original P value obtained through the analysis of the pathway score map; FDR: the error trigger rate; Impact: obtained through topological analysis Out-of-path influence value.

Table S7

KEGG ID of potential metabolic markers (http://www. genome.jp/kegg/compound/) [52] [53]

**Metabolites**	**KEGG ID**	**Metabolites**	**KEGG ID**
Linoleic acid	C01595	D-mannose 1-phosphate	C00636
1-Stearoyl-2-oleoyl-sn-glycerol 3-phosphocholine	C00157	D-Mannose	C00159
Nicotinamide	C00153	Uracil	C00106
L-Aspartate	C00049	L-Arginine	C00062
Thymidine	C00214	Cytidine monophosphate N-acetylneuraminic acid	C00128
Thymine	C00178	Phosphatidylcholine	C00157

Figure S1

Overlapping TIC spectra of negative and positive ion modes of QC samples

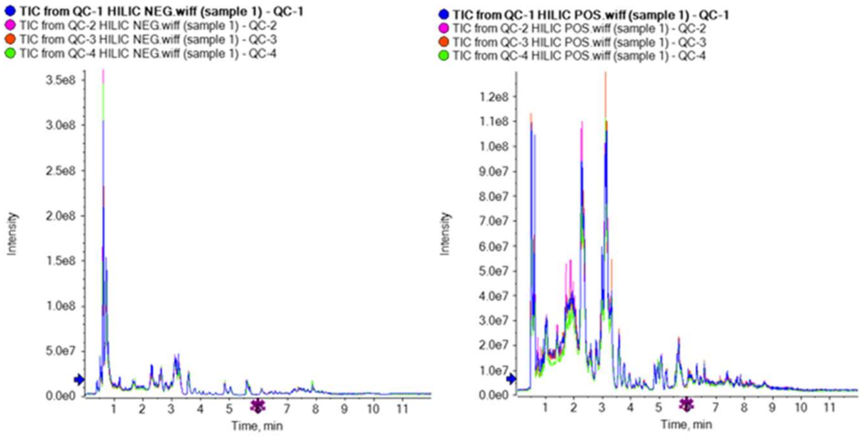

Figure S2

Hotellings T2 diagram in negative and positive ion mode of the sample

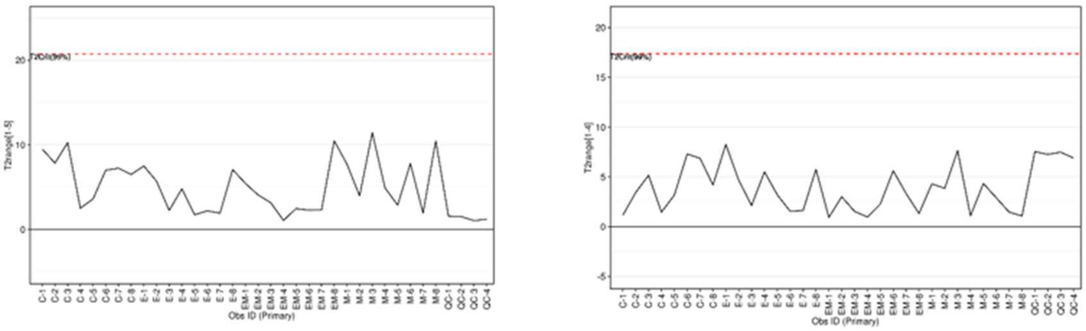

Figure S3

Acute high-intensity exercise metabolic pathways constructed by Met-PA database

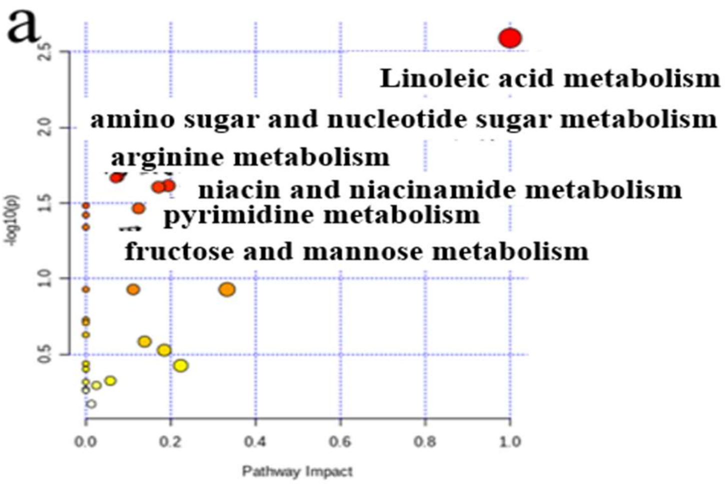

The abscissa pathway impact is the importance value of the metabolic pathway obtained by

topological analysis, and the ordinate -logP is the significance level of the metabolic pathway enrichment analysis; the greater the pathway impact and -logP value are, the higher the correlation of the metabolic differences between different groups is, the bigger the circle is.

Figure S4

Assessment of the diagnostic power of receiver operating characteristic curves for the differential metabolites acute high-intensity exercise.

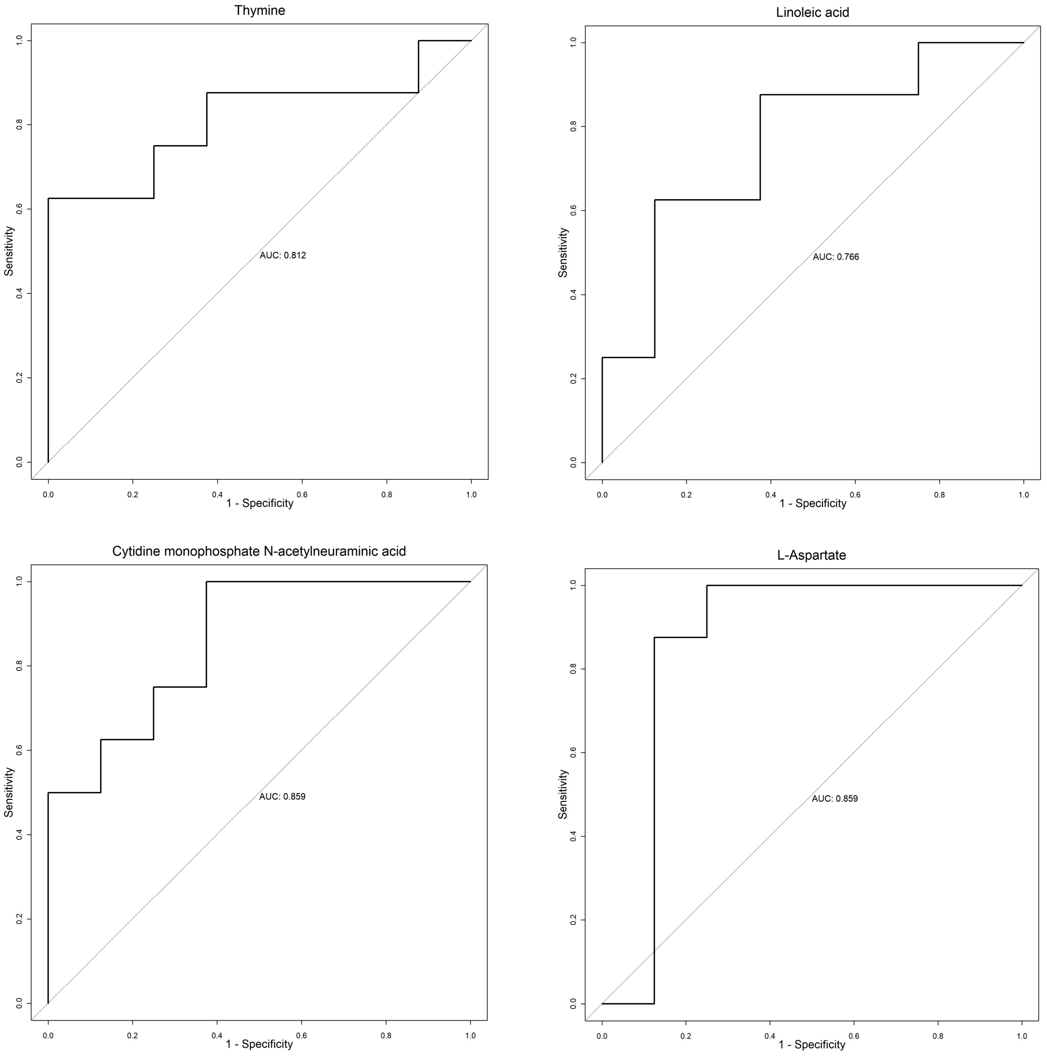

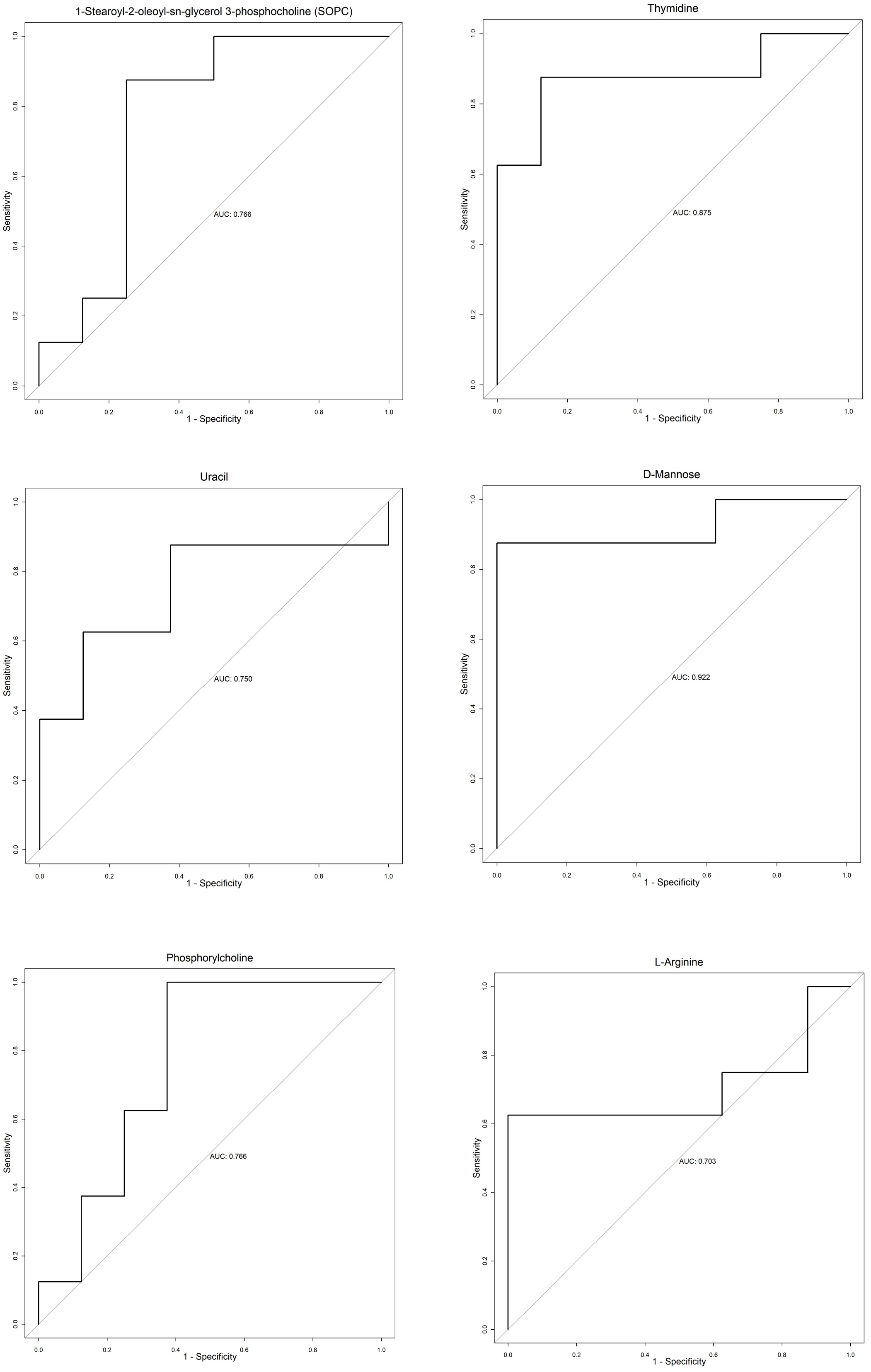
1-Stearoyl-2-oleoyl-sn-glycerol 3-phosphocholine (SOPC)
AUC: 0.766
Thymidine
AUC: 0.875
Uracil
AUC: 0.750
D-Mannose
AUC: 0.922
Phosphorylcholine
AUC: 0.766
L-Arginine
AUC: 0.703
Sensitivity
1 - Specificity

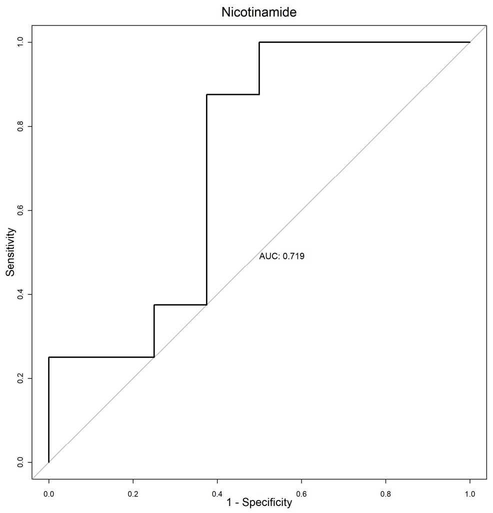

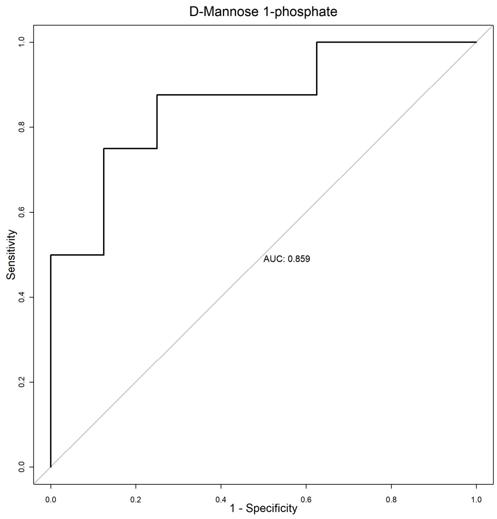

The area under the ROC curve (AUC) can represent the diagnostic effect of markers, and the closer the AUC is to 1, the better the diagnostic effect. AUC>0.9, high accuracy; 0.7<AUC<0.9, some accuracy; 0.5<AUC<0.7, low accuracy; AUC≤0.5, no diagnostic value.
